# Long-Term Survival and Risk Factors in Patients with Hepatitis B-Related Hepatocellular Carcinoma: A Real-World Study

**DOI:** 10.1155/2022/7750140

**Published:** 2022-08-23

**Authors:** Yu Zhu, Ling-Ling Gu, Fa-Biao Zhang, Guo-Qun Zheng, Ting Chen, Wei-Dong Jia

**Affiliations:** ^1^Department of Hepatopancreatobiliary Surgery, Taizhou Hospital of Zhejiang Province Affiliated to Wenzhou Medical University, Taizhou 317000, China; ^2^Division of Liver Surgery, The First Affiliated Hospital of USTC, Division of Life Sciences and Medicine, University of Science and Technology of China, Hefei 230001, China

## Abstract

A retrospective cohort study was conducted to collect 465 patients with hepatitis B-related hepatocellular carcinoma who had undergone radical hepatectomy from January 1, 2012, to August 31, 2018, at the First Affiliated Hospital of the University of Science and Technology of China. The clinical, pathological, and follow-up information was collected to compare the basic characteristics of death and nondeath after radical resection. Kaplan–Meier curves were used for survival analysis and male and female subgroup analysis. The multivariate Cox proportional-hazards regression model was used to analyze independent risk factors related to postoperative death. Of the 465 patients with radical resection of hepatitis B-related hepatocellular carcinoma, 132 died, and 1-, 3-, and 5-year cumulative survival rates after operation were 92.1%, 78%, and 64%, respectively. In the male and female subgroup, 115 and 17 patients died, respectively. The 1-, 3-, and 5-year cumulative survival rates were 92.6%, 77.0%, and 62.6%, respectively, in men, and 89.6%, 78.8%, and 70.2%, respectively, in women. Multivariate Cox proportional-hazards regression analysis showed that microvascular invasion (MVI), Edmondson III/IV, BCLC stage B, and total bilirubin (TB) > 20.5 *μ*mol/L were independent risk factors in patients with hepatitis B-related hepatocellular carcinoma after radical hepatectomy.

## 1. Introduction

Globally, cancer is the second leading cause (the first being cardiovascular disease) of death, liver cancer ranks fourth among the causes behind cancer-related death [1]. The annual incidence of liver cancer cases is 85000 individuals worldwide [2]. The incidence rate of liver cancer is 4.46/1000 in China, and the mortality rate is 4.22/1000 [3]. Hepatocellular carcinoma (HCC) has an annual incidence rate of over 15/100000 in East Asia and Sub-Saharan Africa; 5–15/100000 people in the Mediterranean basin, Southern Europe, and North America, and less than 5/100000 people in Northern Europe [4]. Hepatectomy is the first choice for the treatment of HCC. However, according to the US tumor statistics released in January 2020, the 5-year survival rate of liver cancer in the US is only 18% [5], and the long-term survival rate of HCC patients is not satisfactory. What factors affect the long-term survival of patients with hepatitis B (HBV)-related HCC after operation? Based on real-world data, we retrospectively studied the factors of prognosis after radical resection of HBV-related HCC in patients discharged from January 1, 2012, to August 31, 2018, in the First Affiliated Hospital of the University of Science and Technology of China (USTC) to guide the clinical prevention of HBV-related HCC patients with a high risk of postoperative death, apply positive preventive measures, and improve the survival rate of this group of patients.

## 2. Methods

### 2.1. Patient Population

**Figure 1:**
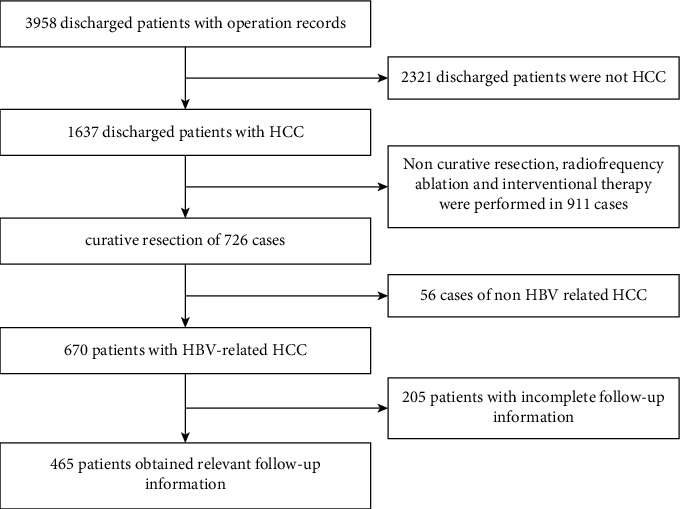
Patients' selection process at the First Affiliated Hospital of USTC.

Through the case data of liver cancer patients in the First Affiliated Hospital of the USTC database initiated and established by the Chinese Society of Clinical Oncology (CSCO), and through the inpatient information management system of the First Affiliated Hospital of USTC, a total of 3958 discharged patients who had undergone liver surgery from January 1, 2012, to August 31, 2018, were selected. According to the postoperative pathological results, 1637 patients with HCC were screened. Furthermore, 465 patients with HBV-related HCC who had undergone radical resection were enrolled in the research. This study was reviewed by the hospital ethics committee. The detailed screening process is described in Figure 1.

### 2.2. Data Collection

The following components were collected: name, gender, age, drinking history, smoking history, prothrombin time (PT), platelet count (PLT), anti-HBc, hepatitis B surface antigen, serum alpha fetoprotein (AFP), HBV-DNA, serum total bilirubin (TB), serum alanine aminotransferase (ALT), serum albumin (ALB), neutrophil/lymphocyte ratio (NLR), platelet/lymphocyte ratio (PLR), serum albumin/globulin ratio (AGR), Child–Pugh grade, operation times, intraoperative bleeding, operation method, hepatectomy method, perioperative blood transfusion, tumor size, tumor number, cirrhosis, microvascular invasion (MVI), ascites, tumor envelope integrity, Edmondson classification, China clinical stage of liver cancer (CNLC) (2017) [6], and Barcelona liver cancer (BCLC) stage (2018) [7]. When there were multiple masses, the tumor diameter was calculated as the sum of the maximum diameters of each mass. When Edmondson's classification of two masses in the same specimen was different, the dominant mass was used for the classification.

### 2.3. Inclusion Criteria Were as Follows

1. Pathologically diagnosed HCC
2. Lack of tumor thrombi in hepatic vein, portal vein, bile duct, and inferior vena cava
3. Lack of invasion of adjacent organs, hilar lymph nodes, and distant metastases
4. Complete tumor tissue specimens
5. Patients on whom only surgical treatment was performed, and no transarterial chemoembolization (TACE), radiofrequency, or microwave treatment was performed before or during operation.

### 2.4. Exclusion Criteria Were as Follows

1. Simultaneous presence of other malignant tumors
2. Perioperative death
3. No history of hepatitis B or hepatitis C virus antibody positivity
4. Patients undergoing nonradical surgery
5. Patients with incomplete pathological and clinical data
6. Lack of follow up for survival

During the follow up, the survival status of patients was checked by telephone interviews. The starting point of the follow up was the operation date, and the ending point was death or December 31, 2019. The survival time was calculated from the date of operation to the date of death or the deadline of follow up, and it was calculated in months. The survivors were treated with truncated values, and the time of death was accurate to year and month.

### 2.5. Statistical Analysis

SPSS 17.0 software was used for statistical analysis. Measurement data satisfying normal or approximately normal distribution were expressed as mean ± SD, and comparisons between groups were carried out by *t*-tests; the median (*M*) (P25∼p75) was used for the statistical description of nonnormal distribution measurement data, and a nonparametric rank sum test was used for intergroup comparisons; the statistical description of categorical variables was carried out by frequencies, and the comparisons between groups were carried out by the chi-square test. The Kaplan–Meier method was used for the survival curves of postoperative death, and a multivariate Cox proportional-hazards regression model was used to analyze the independent risk factors of postoperative death.

## 3. Results

A total of 465 patients with hepatitis B-related HCC after radical resection were followed up for death events up to December 31, 2019. During a median follow up of 34.0 (21.0, 56.0) months, the average age of the patients was 56.42 ± 11.6 years.  Table 1 shows the basic characteristics of patients with hepatitis B-related HCC after radical resection.

**Figure 2:**
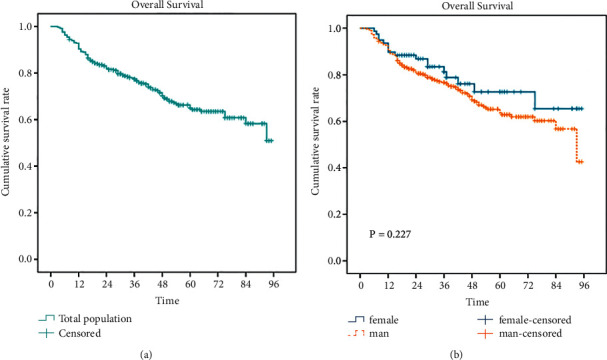
The overall cumulative survival rate of postoperative mortality in patients with hepatitis B-related HCC patients (a). The cumulative survival rate of men and women subgroups (b).

Towards the end of the follow-up period, 132 (28.4%) of them died, and the median survival time was 94 months. As shown in Figure 2(a), the 1-, 3-, and 5-year cumulative survival rates among patients with hepatitis B-related HCC after radical resection were 92.1%, 78%, and 64%, respectively. By the end of follow-up, 115 male HCC patients had died, and the median survival time was 85 months; a total of 17 female HCC patients had death outcome events, and the median survival time was N/A. As shown in Figure 2(b), in men, the 1-, 3-, and 5-year cumulative survival rates were 92.6%, 77.0%, 62.6%, respectively. In women, the 1-, 3-, and 5-year cumulative survival rates were 89.6%, 78.8%, 70.2%, respectively. The log-rank test showed that no significant difference in the survival curve between men and women (*P*=0.227).

**Figure 3:**
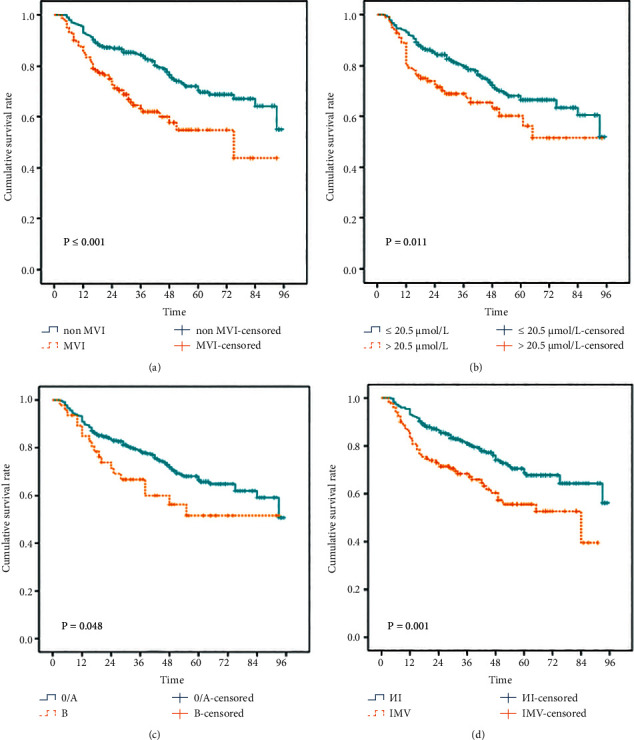
The overall cumulative survival rate stratified by MVI and non-MVI (a), TB ≤ 20.5 *μ*mol/L and TB > 20.5 *μ*mol/L (b), BCLC 0/A and BCLC B (c), and Edmondson I/II and III/IV (d).

As shown in Table 1, NLR, PLR, AGR, intraoperative bleeding, tumor size, MVI, Edmondson stage, preoperative TB level, and BCLC stage were independent risk factors influencing the prognosis of patients with HBV-related HCC after radical resection. With survival outcome (death) and survival time as dependent variables and all independent risk factors as independent variables, multivariate Cox proportional-hazards regression model analysis was established (variable screening method stepwise, variable inclusion criterion *α* ＝ 0.05, and the rejection standard is 0.1). As shown in Table 2, MVI (yes vs. none, hazard ratio (HR) = 2.04, 95%confident interval (CI): 1.42–2.91), Edmondson stage (III/IV vs. I/II, HR = 1.68, 95% CI: 1.18–2.39), BCLC stage (B vs. 0-A, HR = 1.86, 95% CI: 1.14–3.04), and preoperative TB level (>20.5 *μ*mol/L vs. ≤20.5 *μ*mol/L, HR = 1.61, 95% CI: 1.10–2.36) were independent risk factors for postoperative mortality in patients with HBV-related HCC (*P* < 0.05). The albumin/globulin ratio was an independent protective factor for postoperative death in patients with HBV-related HCC (HR = 0.36, 95% CI: 0.19–0.70, *P*=0.0028). As shown in Figure 3, the statistical significance for the overall cumulative survival rate stratified by MVI and non-MVI (Figure 3(a), *P* ≤ 0.001), preoperative TB level ≤20.5 *μ*mol/L and preoperative TB level >20.5 umol/L (Figure 3(b), *P*=0.011), BCLC 0/A and BCLC B (Figure 3(c), *P*=0.048), and Edmondson I/II and III/IV (Figure 3(d), *P*=0.001).

## 4. Discussion

There have been few studies on the risk factors of postoperative death in HBV-related HCC patients based on real-world date. It is generally believed that HCC is caused by chronic liver inflammation due to hepatitis virus infection, alcoholic/nonalcoholic fatty liver, aflatoxin, and other factors. Even after radical resection, patients are still at a high risk of death [8]. How to improve the survival rate of HBV-related HCC patients has always been a problem for liver surgeons. Identification of risk factors affecting the prognosis of patients after radical resection of HBV-related HCC and providing active and effective intervention is needed to improve the survival rate of these patients and the therapeutic effect on HCC.

Different research centers have come to different conclusions about the risk factors affecting survival after radical resection of HCC. Through real-world data, we analyzed 465 patients with HBV-related HCC who had undergone radical resection. The median survival time was 94 months. The 1-, 3-, and 5-year cumulative survival rates were 92.1%, 78%, and 64%, respectively. We found that BCLC staging, MVI, Edmondson staging, and preoperative TB levels were the risk factors affecting postoperative survival in patients with HBV-related HCC.

BCLC is widely used in clinical practice. Patients with very-early and early HCC (BCLC stage 0 and A) should undergo surgical resection, while patients with medium (BCLC stage B) and advanced (BCLC stage C) HCC are recommended to receive TACE or tyrosine kinase inhibitors (TKI) and inhibitors sorafenib or lenvatinib. BCLC mainly considers the tumor number, tumor size and the greater diameter of the tumor, and the number of multiple tumors as factors of a poor prognosis in HCC patients. The increase of the tumor diameter also increases MVI. This was also confirmed by the results, given that the overall survival rate of patients with BCLC stage B was lower than that of patients with BCLC stage 0/A. In addition, some surgeons have suggested that some patients with BCLC stage B HCC could be treated surgically; after selective BCLC stage B patients had been treated surgically, the 5-year overall survival rate of these patients reached 63.4% [9]. In our study, a total of 9 HCC patients who had undergone radical surgery in BCLC stage B were included, and five of them survived and achieved good treatment results. Therefore, BCLC stage B is not a contraindication for surgery. Other studies have shown that TNM stages III and IV also predict a poor prognosis [10].

MVI refers to intravascular tumor invasion that cannot be found in preoperative auxiliary examinations, such as enhanced-computed tomography (CT) or enhanced-magnetic resonance imaging (MRI) and intraoperative HCC tissue specimen examination, it is identified as a microscopic intravascular tumor thrombus during postoperative pathological examination [11]. The incidence of MVI in HCC is 29%–45%, and a larger tumor volume is associated with a higher probability of MVI [12]. MVI is one of the most important pathological indexes to predict the prognosis of HCC resection [13, 14]. However, MVI can only be determined by postoperative pathological examination, so it is difficult to predict before operation. How to accurately predict MVI before operation has become a research hotspot. Recently, (18F) FDG PET/CT radiation histograms were developed to predict the MVI status and disease-free survival in patients with very early and early HCC (BCLC 0 and BCLC A), showing good discrimination and calibration [15]. There was also a study on extreme gradient boosting (XGBoost) and deep learning prediction models based on CT images to predict MVI [16], and these models are expected to predict the probability of MVI before operation, which is conducive to the clinical treatment decision of HCC.

In addition, it has been reported that preoperative liver function is one of the main factors affecting the prognosis of patients [17]. The serum albumin level and serum TB level reflect the synthetic and metabolic functions of the liver and the nutritional status of patients to a certain extent. A recent study has found that the decrease of the preoperative serum albumin level is significantly related to the decrease of the overall survival rate after radical resection of HCC [18]. Li et al. [19] found that the increase in the serum TB level was an independent risk factor for patients' 3-year survival. These findings are similar to our findings, showing that preoperative TB > 20.5 *μ*mol/L was an independent risk factor for death after HBV-related HCC radical resection. In addition, the study in [20] and a previous study by our research group [21] have shown that the ratio of the C reactive protein to albumin is an independent prognostic factor after radical resection of HCC. Our study also found that a higher albumin/globulin ratio was a protective factor in patients after HBV-related HCC radical resection, which confirms the abovementioned theory.

The Edmondson stage represents the differentiation degree of tumor cells, which also reflects the biological characteristics of the tumor. A lower Edmondson stage (stage I/II) is associated with better tumor differentiation, the closer the tumor cells are to normal hepatocytes, the lower is their degree of malignancy, and the smaller is the recurrence probability after surgical resection, and the prognosis is better [22]. A previous study has found that PD-L1 is an independent prognostic factor in patients with liver cancer, and the high expression of PD-L1 significantly correlates with the high Edmondson grade (*P* < 0.01) [23].The N-ethylmaleimide-sensitive fusion protein attachment protein receptor YKT6 and pregnancy upregulated nonubiquitous calmodulin kinase (PNCK) are closely related to the progression HCC, which may be used as potential biomarkers of a poor prognosis in patients with HCC. The expression levels of YKT6 and PNCK in HCC are also related to the Edmondson stage. The higher the stage, the higher the expression of YKT6 and PNCK, and the worse the prognosis of patients [24, 25]. The trauma caused by surgery, the reduction of postoperative immunity, and the acceleration of hepatocyte regeneration after surgery lead to the growth of potential micrometastasis [26], which leads to the early recurrence and a poor prognosis of HCC.

China has a large hepatitis B population. Most of the HCC patients have a chronic hepatitis B and cirrhosis background. Liver cirrhosis leads to the decline of the liver function and affects the volume of the liver tissue removed [27]. The intention to preserve the residual liver volume as much as possible may lead to the residual cancer tissue during operation. In addition, liver cirrhosis is a risk factor for late recurrence after radical resection of HCC [28]. Liver cirrhosis can lead to the recurrence of HCC, which can affect the long-term survival of patients. Liver cirrhosis-related complications, such as portal hypertension and upper gastrointestinal bleeding caused by esophageal and gastric varices also affect the long-term survival of these patients. This study focused on HBV-related HCC patients and explored the factors associated with the prognosis of these patients to improve the survival rate of these patients.

According to our research results, after hepatectomy for hepatitis B-related HCC patients with high-risk factors, the liver function of patients can be improved before operation, and preventive TACE or TKI drugs can be given after operation to prolong the postoperative survival time of these patients. At present, TKI drugs (sorafenib or lenvatinib) combined PD-1/PD-L1 has been used in the treatment of advanced HCC and has achieved some therapeutic effects, but the efficacy of sorafenib is limited by the development of drug resistance. RAF, BRAF, and MEK pathways play a key role and have an impact on the prognosis of patients with advanced HCC. lnc-RNA of BRAF could be another mechanism of cancer proliferation and TKI escape in HCC and the inhibition could become a possible strategy treatment for HCC [29].

There are still some limitations in this study. The sample number was small, and the statistical analysis of all possible risk factors was not carried out. We plan to overcome these issues in further multicenter research.

## Figures and Tables

**Table 1:** Basic characteristics of patients with hepatitis B-related HCC after radical resection (*n* = 465).

Baseline characteristics	Nondied	Died	*P*
Age (years)	56.19 ± 11.27	56.99 ± 12.43	0.502
Sex (number of cases (%))			0.157
Female/male	61 (18.3)/272 (81.7)	17 (12.9)/115 (87.1)	
Drinking (number of cases (%))		/	0.571
No/yes	270 (81.1)/63 (18.9)	110 (83.3)/22 (16.7)	
Smoking (number of cases (%))			0.704
No/yes	238 (71.5)/95 (28.5)	92 (69.7)/40 (30.3)	
NLR	1.9 (1.5, 2.6)	2.4 (1.8.3.5)	0.001
PLR	90.5 (68.6, 124.3)	104.5 (78.0, 146.5)	0.002
AGR	1.43 ± 0.27	1.36 ± 0.26	0.008
Intraoperative bleeding (ml)	200 (100, 300)	225 (100.425)	0.001
Tumor size (number of cases (%))			0.002
≤5/>5 cm	172 (51.6)/161 (48.4)	47 (35.6)/85 (64.4)	
Cirrhosis (number of cases (%))			0.927
No/yes	77 (23.1)/256 (76.9)	30 (22.7)/102 (77.8)	
MVI (number of cases (%))			0.011
None/yes	228 (68.5)/105 (31.5)	74 (56.1)/58 (43.9)	
Number of tumors (number of cases (%))			0.158
1/≥2	296 (88.9)/37 (11.1)	111 (84.1)/21 (15.9)	
Intact capsule (number of cases (%))			0.993
No/yes	68 (20.4)/265 (79.6)	27 (20.5)/105 (79.5)	
Anatomical resection (number of cases (%))			0.802
No/yes	246 (73.9)/87 (26.1)	99 (75.0)/33 (25.0)	
AFP (number of cases (%))			0.151
<400/≥400 ng/ml	230 (69.1)/103 (30.9)	82 (62.1)/50 (37.9)	
Edmondson (number of cases (%))			0.002
I and II/III and IV	253 (76.0)/80 (24.0)	81 (61.4)/51 (38.6)	
Anti-HBc (number of cases (%))			0.881
Positive/negative	322 (96.7)/11 (3.3)	128 (97.0)/4 (3.0)	
HBsAg (number of cases (%))			0.657
Positive/negative	283 (85.0)/50 (15.0)	110 (83.3)/22 (16.7)	
HBV-DNA (number of cases (%))			0.057
<1000/≥1000 copies/ml	135 (40.5)/198 (59.5)	41 (31.1)/91 (68.9)	
ALT (number of cases (%))			0.369
≤50/>50 U/L	253 (76.0)/80 (24.0)	95 (72.0)/37 (28.0)	
ALB (number of cases (%))			0.184
≥35/<35 g/L	310 (93.1)/23 (6.9)	118 (89.4)/14 (10.6)	
TB (number of cases (%))			0.030
≤20.5/>20.5 *μ*mol/L	268 (80.5)/65 (19.5)	94 (71.2)/38 (28.8)	
PT (number of cases (%))			0.113
≤14/>14 s	312 (93.7)/21 (6.3)	118 (89.4)/14 (10.6)	
Ascites (number of cases (%))			0.235
No/yes	287 (86.2)/46 (13.8)	108 (81.8)/24 (18.2)	
Perioperative blood transfusion (number of cases (%))			0.054
None/yes	278 (83.5)/55 (16.5)	100 (75.8)/32 (24.2)	
Laparoscope (number of cases (%))			0.164
Yes/no	40 (12.0)/293 (88.0)	10 (7.6)/122 (92.4)	
Operation duration (number of cases (%))			0.798
>120/≤120 min	186 (55.9)/147 (44.1)	72 (54.6)/60 (45.4)	
Child–Pugh (number of cases (%))			0.064
A/B	333 (100.0)/0 (0.0)	130 (98.9)/2 (1.5)	
CNLC (number of cases (%))			0.054
Ia/Ib/IIa/IIb	187 (56.2)/112 (33.6)/30 (9.0)/4 (1.2)	53 (40.2)/60 (45.4)/15 (11.4)/4 (3.0)	
BCLC (number of cases (%))			0.015
0/A/B	27 (8.1)/278 (83.5)/28 (8.4)	3 (2.3)/110 (83.3)/19 (14.4)	

**Table 2:** Multivariate Cox regression analysis of mortality risk after curative resection of hepatitis B-related HCC

Risk factors	*n*	HR (95% CI)	*P*
NLR	465	1.05 (1.02–1.11)	0.233
AGR	465	0.36 (0.19–0.70)	0.003
PLR	465	2.11 (0.61–1.34)	0.740
Intraoperative bleeding	465	2.10 (1.34–3.30)	0.839
MVI			0.000
None	302	1	
Yes	163	2.04 (1.42–2.91)	
Tumor size			0.078
≤5 cm	242	1	
>5 cm	223	1.02 (1.10–2.23)	
BCLC			0.013
0-A	418	1	
B	47	1.86 (1.14–3.04)	
Edmondson			0.005
I/II	334	1	
III/IV	131	1.68 (1.18–2.39)	
Preoperative TB level			0.016
≤20.5 *μ*umol/L	362		
>20.5 *μ*mol/L	103	1.61 (1.10–2.36)	

## Data Availability

The data used to support the findings of this study are available from the corresponding author upon request.

## References

[1] GBD 2015 Mortality and Causes of Death Collaborators (2016). Global, regional, and national life expectancy, all-cause mortality, and cause-specific mortality for 249 causes of death, 1980–2015: a systematic analysis for the global burden of disease study 2015. *Lancet*.

[2] Ng K. K., Cheung T. T., Pang H. H. (2019). A simplified prediction model for early intrahepatic recurrence after hepatectomy for patients with unilobar hepatocellular carcinoma without macroscopic vascular invasion: an implication for adjuvant therapy and postoperative surveillance. *Surgical Oncology*.

[3] Chen W., Zheng R., Baade P. D (2016). Cancer statistics in China, 2015. *CA: A Cancer Journal for Clinicians*.

[4] Ferlay J., Soerjomataram I., Dikshit R. (2015). Cancer incidence and mortality worldwide: sources, methods and major patterns in GLOBOCAN 2012. *International Journal of Cancer*.

[5] Siegel R. L., Miller K. D., Jemal A. (2020). Cancer statistics, 2020. *CA: A Cancer Journal for Clinicians*.

[6] Zhou J., Sun H. C., Wang Z. (2018). Guidelines for diagnosis and treatment of primary liver cancer in China (2017 edition). *Liver Cancer*.

[7] European Association for the Study of the Liver (2018). EASL clinical practice guidelines: management of hepatocellular carcinoma. *Journal of Hepatology*.

[8] Bertuccio P., Turati F., Carioli G. (2017). Global trends and predictions in hepatocellular carcinoma mortality. *Journal of Hepatology*.

[9] Wada H., Eguchi H., Noda T. (2016). Selection criteria for hepatic resection in intermediate-stage (BCLC stage B) multiple hepatocellular carcinoma. *Surgery*.

[10] Li M., Wang Z., Cao J. (2019). Risk factors and prognosis of patients with recurrent hepatocellular carcinoma who undergo liver re-resections. *European Journal of Surgical Oncology*.

[11] Jin Y., Li J. T. (2013). Research progress in related clinical factors and molecular biomarkers for microvascular invasion in hepatocellular carcinoma. *Journal of Clinical Hepatology*.

[12] Hirokawa F., Hayashi M., Miyamoto Y. (2015). Predictors of poor prognosis by recurrence patterns after curative hepatectomy for hepatocellular carcinoma in child-Pugh classification A. *Hepato-Gastroenterology*.

[13] Kobayashi T., Aikata H., Kobayashi T., Ohdan H., Arihiro K., Chayama K. (2017). Patients with early recurrence of hepatocellular carcinoma have poor prognosis. *Hepatobiliary and Pancreatic Diseases International*.

[14] Chan A. C., Fan S. T., Poon R. T. (2013). Evaluation of the seventh edition of the American Joint Committee on Cancer tumour-node-metastasis (TNM) staging system for patients undergoing curative resection of hepatocellular carcinoma: implications for the development of a refined staging system. *International Hepato-Pancreato-Biliary Association*.

[15] Li Y., Zhang Y., Fang Q. (2021). Radiomics analysis of (18F)FDG PET/CT for microvascular invasion and prognosis prediction in very-early- and early-stage hepatocellular carcinoma. *European Journal of Nuclear Medicine and Molecular Imaging*.

[16] Jiang Y. Q., Cao S. E., Cao S. (2021). Preoperative identification of microvascular invasion in hepatocellular carcinoma by XGBoost and deep learning. *Journal of Cancer Research and Clinical Oncology*.

[17] Toyoda H., Lai P. B., O’Beirne J. (2016). Long-term impact of liver function on curative therapy for hepatocellular carcinoma: application of the ALBI grade. *British Journal of Cancer*.

[18] Wang L., Li Q., Zhang J., Lu J. (2019). A novel prognostic scoring model based on albumin and *γ*-glutamyltransferase for hepatocellular carcinoma prognosis. *Cancer Management and Research*.

[19] Li J., Zhu W. L., Kang X. X. (2017). Prognostic factors and model of primary liver cancer treated with transcatheter arterial chemoembolization combined with radiofrequency ablation. *Zhonghua Zhongliu Zazhi*.

[20] Ren Y., Fan X., Chen G., Zhou D., Lin H., Cai X. (2019). Preoperative C-reactive protein/albumin ratio to predict mortality and recurrence of patients with hepatocellular carcinoma after curative resection. *Medicina Clínica*.

[21] Du X. F., Xu Y. F., Dai Q. Q. (2019). Correlation of C-reaction protein/albumin ratio with pathological feature and prognosis of primary liver cancer. *Chinese Journal of Health Laboratory Technology*.

[22] Peng Z., Wei M., Chen S. (2018). Combined transcatheter arterial chemoembolization and radiofrequency ablation versus hepatectomy for recurrent hepatocellular carcinoma after initial surgery: a propensity score matching study. *European Radiology*.

[23] Chen L., Huang X., Zhang W. (2020). Correlation of PD-L1 and SOCS3 Co-expression with the prognosis of hepatocellular carcinoma patients. *Journal of Cancer*.

[24] Xu J. Z., Jiang J. J., Xu H. J., Sun X. D., Liu Z. C., Hu Z. M. (2021). High expression of YKT6 associated with progression and poor prognosis of hepatocellular carcinoma. *Scandinavian Journal of Gastroenterology*.

[25] Cho Y. A., Choi S., Park S., Park C. K., Ha S. Y. (2020). Expression of pregnancy up-regulated non-ubiquitous calmodulin kinase (PNCK) in hepatocellular carcinoma. *Cancer Genomics &Proteomics*.

[26] Yang L., Yuan Z. Y., Zhang Y. M. (2019). Research progress of the relationship between liver regeneration and tumor recurrence after hepatectomy. *Zhonghua Wai Ke Za Zhi*.

[27] Zhu Y., Li Z. Y., Wang C. G., Fang Z. P., Jia W. D., Zhang F. B. (2020). Laparoscopic combined with thoracoscopic transdiaphragmatic hepatectomy for hepatitis B-related hepatocellular carcinoma located in segment VII or VIII. *Hepatobiliary and Pancreatic Diseases International*.

[28] Cheng Z., Yang P., Qu S. (2015). Risk factors and management for early and late intrahepatic recurrence of solitary hepatocellular carcinoma after curative resection. *International Hepato-Pancreato-Biliary Association*.

[29] Gnoni A., Licchetta A., Memeo R. (2019). Role of BRAF in hepatocellular carcinoma: a rationale for future targeted cancer therapies. *Medicina*.

